# Long-Tail Aware Cross-Modal Graph Attention Network for Fine-Grained Indoor 3D Semantic Segmentation of Point Clouds

**DOI:** 10.3390/s26113401

**Published:** 2026-05-27

**Authors:** Erdal Özbay, Feyza Altunbey Özbay

**Affiliations:** 1 Department of Computer Engineering, Firat University, Elazig 23119, Türkiye; 2 Department of Software Engineering, Firat University, Elazig 23119, Türkiye; faltunbey@firat.edu.tr

**Keywords:** cross-modal fusion, graph attention networks, long-tail learning, point cloud, semantic segmentation

## Abstract

**Highlights:**

**What are the main findings?**

- The proposed LT-CM-GACNet++ effectively improves semantic segmentation performance on long-tail distributed indoor point cloud data by leveraging cross-modal feature fusion.
- The integration of CMGA and prototype-based long-tail learning significantly enhances rare-class recognition while maintaining strong accuracy.

**What are the implications of the main findings?**

- The results demonstrate that cross-modal learning combined with long-tail aware optimization provides a robust solution for fine-grained 3D indoor scene understanding.
- The proposed framework can be extended to other multimodal 3D vision tasks, offering a scalable approach for handling class imbalance in large-scale real-world datasets.

**Abstract:**

Accurate and efficient semantic segmentation of point cloud data is critical in many application areas involving indoor scene understanding. In particular, fine-grained object categories, high data density, and class imbalance in high-resolution indoor datasets significantly limit class discrimination in 3D semantic segmentation. The multimodal data structure, high-fidelity geometry, and long-tail class distribution of the recently popular ScanNet++ dataset further exacerbate these challenges. This study proposes a novel Long-Tail Aware Cross-Modal Graph Attention Network (LT-CM-GACNet++) to address fine-grained 3D semantic segmentation under long-tail distributions. The proposed method integrates dynamic graph-based geometric feature extraction with a lightweight visual feature extractor based on MobileNetV3, enabling effective fusion of geometric and RGB-based information. The proposed Cross-Modal Graph Attention (CMGA) module facilitates adaptive information transfer between modalities, enabling more effective representation learning of both local and global contextual features. To mitigate the adverse effects of long-tail class distributions, prototype-based representation learning and a class frequency-aware loss function are jointly employed. This strategy improves the learning of rare classes while enhancing the discrimination between visually and geometrically similar categories. In the preprocessing stage, density-based sampling, normal vector estimation, and block-based fixed-size point cloud generation are applied to high-resolution mesh-derived data. The proposed model is evaluated on 50 scenes and 100 semantic classes selected from the ScanNet++ dataset. Experimental results demonstrate that the proposed method achieves significant improvements over existing approaches in terms of both overall segmentation performance and rare-class performance. In particular, notable gains are observed in mean Intersection over Union (mIoU) and rare-class mIoU metrics. These results highlight the effectiveness of cross-modal learning for high-resolution 3D scene segmentation under long-tail distributions.

## 1. Introduction

Recent advances in three-dimensional (3D) sensing technologies have significantly expanded the applicability of point cloud data in various domains, including indoor scene understanding, robotics, augmented reality, and digital twin systems [1]. Modern acquisition systems, such as LiDAR scanners and RGB-D cameras, enable the capture of high-resolution geometric and visual information, producing dense and information-rich point clouds. These representations have become a fundamental data modality for understanding complex indoor environments [2].

Semantic segmentation of point cloud data, which involves assigning a semantic label to each point, is a critical task for extracting meaningful information from 3D scenes [3]. Accurate segmentation enables intelligent systems to interpret spatial structures and object relationships, facilitating applications such as scene reconstruction, robotic navigation, and human environment interaction [4]. However, point cloud data inherently exhibit irregular structures, non-uniform density distributions, and high dimensionality, making the segmentation task computationally challenging [5].

In recent years, deep learning-based approaches have significantly improved the performance of point cloud segmentation. Early methods, such as PointNet and its hierarchical extension PointNet++, introduced point-wise feature learning mechanisms [6]. Subsequent graph-based models, including DGCNN, further enhanced local feature representation by modeling relationships between neighboring points. More recently, transformer-based architectures such as Point Transformer have demonstrated strong capabilities in capturing long-range dependencies and contextual information through attention mechanisms [7,8]. Despite these advancements, existing methods still face limitations when applied to high-resolution indoor datasets [9]. The emergence of high-fidelity datasets such as ScanNet++ has introduced new challenges for 3D semantic segmentation [10]. Unlike earlier datasets, ScanNet++ provides dense geometric reconstructions, high-resolution RGB imagery, and a large number of fine-grained semantic categories. In particular, the long-tail distribution of classes where a small number of dominant classes coexist with many underrepresented categories poses a significant challenge for learning robust feature representations. Additionally, visually and geometrically similar classes (e.g., “cabinet”, “kitchen cabinet”, “storage cabinet”) further complicate class discrimination.

Another important limitation of existing approaches is the insufficient exploitation of multimodal information. While RGB features can provide complementary texture and appearance cues, many methods rely on simple feature concatenation strategies, failing to capture complex interactions between geometric and visual modalities [11]. Furthermore, most current models do not explicitly address the long-tail problem, leading to suboptimal performance on rare classes. To address these challenges, this study proposes a novel Long-Tail Aware Cross-Modal Graph Attention Network (LT-CM-GACNet++) for fine-grained indoor 3D semantic segmentation [12]. The proposed framework integrates dynamic graph-based geometric feature learning with a lightweight visual feature extractor based on MobileNetV3, enabling efficient and effective multimodal representation learning. A Cross-Modal Graph Attention (CMGA) module is introduced to facilitate adaptive information exchange between modalities, allowing the model to capture both local geometric structures and global contextual relationships [13]. In addition, a long-tail aware learning strategy is incorporated through prototype-based representation learning and a class frequency-aware loss function [14]. This design improves the recognition of rare classes while enhancing the discrimination between fine-grained categories. The proposed approach is evaluated on a subset of ScanNet++ consisting of 50 scenes and 100 semantic classes, demonstrating its effectiveness in handling high-resolution and long-tail distributed 3D data. The main contributions of this work can be summarized as follows:

- A novel Long-Tail Aware Cross-Modal Graph Attention Network (LT-CM-GACNet++) is proposed for fine-grained indoor 3D semantic segmentation, enabling effective fusion of geometric and RGB-based features through the proposed Cross-Modal Graph Attention (CMGA) mechanism.
- A long-tail learning strategy combining prototype-guided representation learning and class-frequency-aware optimization is introduced to improve feature discrimination and segmentation performance for underrepresented semantic categories.
- A comprehensive experimental evaluation is conducted on a subset of the ScanNet++ dataset containing high-resolution indoor scenes and approximately 100 semantic classes.

The primary methodological contribution of the proposed LT-CM-GACNet++ framework lies in the unified integration of geometry-guided cross-modal attention and prototype-based long-tail optimization within a single end-to-end segmentation architecture. Rather than introducing isolated standalone modules, the proposed framework is specifically designed to jointly address multimodal feature interaction and severe class imbalance in fine-grained indoor 3D semantic segmentation. The coordinated interaction between dynamic graph-based geometric learning, lightweight RGB feature extraction, and frequency-aware prototype regularization enables improved discrimination of visually and geometrically similar minority classes under long-tail distributions.

## 2. Related Works

Efficient processing of point cloud data has attracted significant attention due to its critical role in applications such as indoor scene understanding, robotics, and augmented reality. While substantial progress has been made in 3D semantic segmentation, existing approaches often struggle with challenges such as irregular data structures, class imbalance, and limited utilization of multimodal information. This section reviews recent developments in point cloud segmentation, attention-based models, multimodal fusion, and long-tail learning strategies.

Early deep learning-based approaches for point cloud segmentation, such as PointNet, introduced direct processing of unordered point sets using symmetric functions [15]. This was later extended by PointNet++, which incorporated hierarchical feature learning to capture local geometric structures [16]. Graph-based methods, including DGCNN, further improved segmentation by dynamically modeling relationships between neighboring points through edge convolutions [17]. Despite their effectiveness, these methods often exhibit limited capability in capturing long-range dependencies and are sensitive to non-uniform point distributions. To overcome these limitations, transformer-based architectures have recently been introduced for point cloud processing. Models such as Point Transformer leverage self-attention mechanisms to capture global contextual relationships [18]. Subsequent works, including stratified transformers and Point Transformer v3, have improved scalability and efficiency by optimizing attention computation and receptive field design [19,20]. Additionally, hybrid attention mechanisms combining spatial and channel-wise attention have been explored to enhance feature representation. For instance, Squeeze-and-Excitation (SE) blocks enable adaptive channel recalibration by emphasizing informative features while suppressing noise [21]. However, most existing methods apply these attention mechanisms independently, without fully exploiting their complementary strengths.

Another critical limitation in the current literature is the insufficient integration of multimodal information. While RGB data provides rich appearance cues, many approaches rely on simple feature concatenation strategies, which fail to model complex interactions between geometric and visual modalities. Recent studies have explored cross-modal fusion techniques to address this issue, demonstrating that attention-based fusion mechanisms can significantly improve segmentation performance by aligning complementary features across modalities [22,23]. Nevertheless, these approaches often overlook the challenges introduced by high-resolution datasets and fine-grained semantic categories.

Recent pioneering studies have further explored visual reasoning and introspective learning strategies to enhance representation learning under limited-data conditions. In this context, Wang et al. introduced an introspective feature reasoning mechanism for few-shot semantic segmentation in 3D point clouds [24]. Such approaches highlight the growing importance of semantic-aware representation learning beyond purely geometric feature extraction, particularly for rare-class understanding and limited-sample learning scenarios.

Prototype-guided representation learning has recently emerged as an effective strategy for improving feature discrimination in 3D semantic segmentation under class imbalance conditions. He et al. [25] proposed a representative prototype memorization framework that learns class-aware embeddings for semantic and instance segmentation in RGB-D indoor scenes. Unlike memory-intensive prototype repositories, the proposed LT-CM-GACNet++ framework employs lightweight batch-level prototype regularization integrated with class-frequency-aware optimization, making it computationally more suitable for dense indoor point cloud segmentation.

Recent studies have increasingly focused on addressing long-tail distributions in 3D semantic segmentation through prototype-guided learning, feature memory mechanisms, and adaptive loss weighting strategies. Prototype-based approaches improve minority-class representation by enforcing intra-class compactness and inter-class separability in the feature space. Unlike existing long-tail segmentation methods that mainly rely on geometry-only representations, the proposed LT-CM-GACNet++ framework jointly integrates multimodal cross-modal attention and prototype-aware learning within a unified architecture. This combination enables more robust discrimination of visually and geometrically similar rare classes under highly imbalanced indoor scene distributions.

The emergence of ScanNet++ has introduced new challenges in 3D semantic segmentation. Compared to earlier datasets, ScanNet++ provides high-fidelity geometry, dense reconstructions, and a large number of fine-grained semantic classes. A key characteristic of such datasets is the long-tail distribution of classes, where a small number of dominant categories coexist with a large number of underrepresented ones. This imbalance significantly degrades model performance on rare classes and remains an open problem in 3D vision [10]. To address class imbalance, various strategies have been proposed, including class-balanced loss functions, data augmentation, and oversampling techniques. Focal loss and its variants have been widely adopted to emphasize hard and minority samples [26]. More recently, prototype-based learning approaches have been introduced to improve feature separability for rare classes by guiding representation learning toward class-specific feature centers [27]. While these methods have shown promising results, they are rarely integrated with multimodal and attention-based frameworks in a unified manner.

Despite these advancements, several research gaps remain. First, existing methods often treat geometric learning, multimodal fusion, and long-tail handling as separate problems, leading to suboptimal performance in complex real-world scenarios [28]. Second, many models fail to effectively capture both local geometric structures and global contextual relationships simultaneously [29]. Third, the challenges posed by high-resolution and fine-grained datasets such as ScanNet++ are not sufficiently addressed in current segmentation frameworks. To overcome these limitations, this study proposes a Long-Tail Aware Cross-Modal Graph Attention Network (LT-CM-GACNet++), which integrates dynamic graph-based feature learning, cross-modal attention mechanisms, and prototype-guided long-tail learning within a unified framework. Unlike existing approaches, the proposed method jointly models geometric and visual information while explicitly addressing class imbalance, resulting in improved performance for both dominant and rare classes in high-resolution indoor scenes.

## 3. Materials and Methods

In this study, we propose an end-to-end framework for fine-grained 3D semantic segmentation that jointly addresses four fundamental challenges in point cloud understanding: deep learning-based segmentation, attention modeling, multimodal fusion, and long-tail learning. Unlike conventional approaches that treat these components independently, the proposed framework integrates them into a unified architecture to enhance both representation capacity and generalization [30].

**Figure 1:**
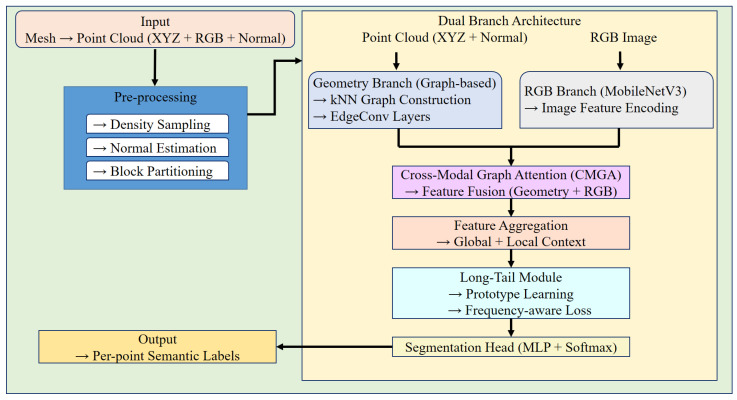
Overall workflow of the proposed LT-CM-GACNet++ framework. The pipeline consists of preprocessing, geometric feature extraction, RGB feature encoding using MobileNetV3, cross-modal graph attention fusion, long-tail aware representation learning, and semantic segmentation prediction.

From a segmentation perspective, dynamic graph-based feature learning is employed to effectively capture local geometric structures in irregular point clouds. To improve feature discrimination, an attention-driven design is incorporated, where both spatial relationships and cross-modal dependencies are adaptively modeled [31]. In addition, multimodal fusion is achieved by integrating geometric features with RGB information through a dedicated cross-modal attention mechanism, enabling complementary feature learning [32]. Finally, to address the long-tail distribution problem inherent in high-resolution indoor datasets such as ScanNet++, a prototype-guided learning strategy combined with a class frequency-aware loss function is introduced [33]. The overall framework is designed to operate in an end-to-end manner, starting from raw mesh-derived point clouds and producing per-point semantic predictions. The proposed framework for semantic segmentation of point cloud is shown in Figure 1.

The pipeline starts with high-resolution mesh-derived point clouds, which are inherently noisy, dense, and non-uniform. A dedicated preprocessing stage is applied to normalize spatial distribution and extract geometric priors. The processed data is then fed into a dual-branch architecture, where geometric features are learned through graph-based operations, and visual features are extracted using a lightweight convolutional backbone. These features are fused using a Cross-Modal Graph Attention (CMGA) mechanism, which dynamically learns the importance of each modality. Finally, a long-tail aware learning strategy is applied to mitigate class imbalance by reweighting the loss function and guiding feature learning through class prototypes.

### 3.1. Dataset

The proposed framework is evaluated on the ScanNet++ dataset, which represents one of the most advanced benchmarks for indoor 3D scene understanding [10]. ScanNet++ extends previous datasets by providing high-resolution 3D reconstructions, dense geometric representations, and aligned RGB data, enabling detailed analysis of complex indoor environments. Unlike earlier datasets, ScanNet++ includes fine-grained semantic annotations with a large number of object categories. A defining characteristic of this dataset is its long-tail distribution, where a limited number of dominant classes (e.g., wall, floor) coexist with numerous underrepresented categories (e.g., stapler, mouse, socket). This imbalance poses substantial challenges for learning robust segmentation models, often resulting in biased decision boundaries and a tendency to overfit dominant classes. Additionally, the presence of fine-grained categories with high inter-class similarity increases intra-class confusion. Therefore, the dataset not only tests segmentation accuracy but also evaluates the robustness of representation learning under challenging distributions. In this study, a subset of 50 scenes is utilized, containing approximately 100 semantic categories. Each scene consists of high-density point clouds derived from mesh reconstructions, with associated RGB values and geometric features such as surface normals. Detailed information about the ScanNet++ dataset is given in Table 1.

### 3.2. Pre-Processing

The proposed framework operates on point clouds uniformly sampled from reconstructed ScanNet++ meshes rather than directly using the raw RGB-D point observations. The preprocessing phase constitutes the first operational block of the proposed framework, as shown in Figure 1, and plays a critical role in transforming raw mesh-derived point cloud data into a structured and learnable representation. High-resolution indoor point clouds are inherently characterized by non-uniform density distributions, sensor-induced noise, and irregular spatial sampling, which can significantly degrade the performance of deep learning models if not properly addressed. To overcome these challenges, the proposed preprocessing pipeline is designed around three complementary operations: density-aware sampling, normal vector estimation, and block partitioning. These steps collectively ensure that the input data is geometrically informative, spatially balanced, and computationally tractable.

#### 3.2.1. Density-Aware Sampling

Point clouds derived from indoor scenes typically exhibit strong density variations due to occlusions, scanning angles, and reconstruction artifacts [34]. For example, planar surfaces such as walls and floors contain excessively dense point distributions, whereas small objects (e.g., keyboard, bottle) are often sparsely represented. This imbalance leads to biased feature learning, where dominant regions disproportionately influence model updates. To address this issue, we define the local density around each point $p_{i}$ using Equation (1):

(1) $$\rho_{i}=\frac{k}{\sum_{j\in N_{k}(i)}\left\|\left(p_{i}-p_{j}\right)\right\|}\;,$$

here, the denominator captures the average distance to neighboring points. A smaller average distance implies a higher local density, while larger distances indicate sparse regions. Based on Equation (1), a density-aware sampling strategy is applied:

- Points located in high-density regions are probabilistically downsampled to reduce redundancy.
- Points in low-density regions are preserved to maintain structural completeness.

This adaptive sampling process ensures that the resulting point cloud achieves a balanced spatial distribution, preventing over-representation of dominant structures while preserving fine-grained details. Consequently, the model is exposed to a more uniform training signal, improving both convergence stability and segmentation accuracy.

#### 3.2.2. Normal Vector Estimation

Raw point coordinates encode positional information but lack explicit local geometric structure. To enrich the representation, surface normal vectors are estimated using local neighborhood statistics [35]. For each point $p_{i}$, the covariance matrix of its *k*-nearest neighbors is computed in Equation (2):

(2) $$C_{i}=\frac{1}{k}\sum_{j\in N_{k}(i)}\left(p_{j}-{\bar{p}}_{i}\right){\left(p_{j}-{\bar{p}}_{i}\right)}^{T},$$

where ${\bar{p}}_{i}$ denotes the centroid of the local neighborhood. Eigenvalue decomposition of $C_{i}$ yields the principal directions of variation, and the eigenvector associated with the smallest eigenvalue is selected as the surface normal. Normal vectors capture local surface orientation and improve discrimination between geometrically distinct structures. Each point is therefore represented in Equation (3):

(3) $$p_{i}=\left(x_{i},y_{i},z_{i},n_{i}\right),$$

This enriched representation provides more informative geometric features for subsequent learning stages.

#### 3.2.3. Block Partitioning

Given the large scale of indoor scenes (often containing millions of points), directly processing the entire point cloud is computationally infeasible. To address this limitation, the scene is partitioned into smaller, fixed-size spatial blocks [36]. Blocks are generated with a fixed spatial size of 1.5 m × 1.5 m × 1.5 m and a stride of 0.75 m between adjacent windows, resulting in a 50% overlap ratio. Each block is defined as in Equation (4):

(4) $$P_{b}=\{{p_{i}\}}_{i=1}^{1024},$$

This fixed-size representation ensures compatibility with batch-based deep learning frameworks and enables efficient GPU utilization. The partitioning process follows these steps:

1. The scene is divided into overlapping spatial regions.
2. For each region:
  - If the number of points exceeds 1024, random sampling is applied.
  - If the number of points is insufficient, zero-padding is introduced.

This strategy ensures consistent input dimensionality across all samples, which is essential for stable training. Additionally, block-based processing allows the model to focus on local spatial context, while global context is later reconstructed through feature aggregation.

### 3.3. Proposed LT-CM-GACNet++ Model Architecture

**Figure 2:**
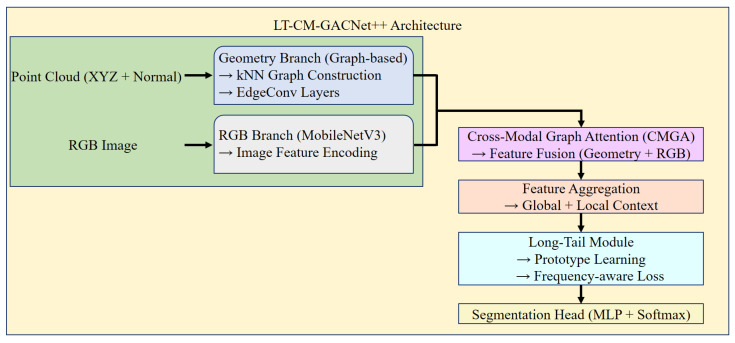
Internal architecture of the proposed LT-CM-GACNet++ model. The framework integrates EdgeConv-based geometric feature learning with RGB embeddings extracted from MobileNetV3-small. The proposed CMGA module adaptively fuses multimodal representations before hierarchical context aggregation and segmentation prediction.

The internal architecture of the proposed LT-CM-GACNet++ model, as shown in Figure 2, follows a dual-branch design, where geometric and visual features are extracted independently and subsequently fused through an attention-driven mechanism. This design is motivated by the observation that:

- Geometric features capture spatial structure and shape information.
- RGB features encode appearance, texture, and color cues.

By decoupling these modalities in the early stages and integrating them in later stages, the model learns more robust and discriminative representations for fine-grained 3D semantic segmentation through effective fusion of geometric and visual information.

The geometric branch operates directly on point cloud data using dynamic graph construction to capture local spatial relationships. Edge-based feature learning is employed to encode geometric structures through neighborhood aggregation. In parallel, the RGB branch utilizes MobileNetV3 to extract high-level visual features from aligned image patches. These two feature streams are then integrated through the proposed Cross-Modal Graph Attention (CMGA) module, which adaptively learns interactions between geometric and visual modalities. For each point $p_{i}$ the RGB frame with the highest visibility score and minimum viewing angle deviation is selected. Unlike conventional fusion strategies based on concatenation, the proposed architecture leverages attention-based weighting to dynamically prioritize informative features across modalities. The fused representation is subsequently refined through hierarchical feature aggregation and passed to the segmentation head for per-point classification.

To establish the correspondence between 3D points and RGB observations, each point ($p_{i}\;\epsilon \;R^{3}$) is projected into candidate RGB frames using the camera intrinsic and extrinsic parameters provided by the ScanNet++ dataset. Since a single 3D point may be visible in multiple RGB views due to multi-view reconstruction, a view selection strategy is applied to determine the most representative observation.

For each point, all valid projections are first identified based on visibility constraints and image boundary conditions. Among the candidate views, the RGB frame with the minimum reprojection distance to the image center is selected. This strategy reduces perspective distortion and minimizes partial occlusion effects commonly observed near image boundaries. After view selection, a fixed-size local image patch centered around the projected pixel coordinate is extracted and fed into the MobileNetV3 branch for visual feature encoding.

The extracted RGB embeddings are subsequently aligned with the corresponding geometric point features through the proposed Cross-Modal Graph Attention (CMGA) module. In the current implementation, feature extraction is performed using a single-view strategy without explicit multi-view feature aggregation. Although multi-view fusion may further enhance contextual consistency, the adopted strategy provides an efficient trade-off between computational complexity and multimodal representation quality.

In the proposed architecture, dynamic graph construction is performed using *k* = 16 nearest neighbors to capture local geometric relationships. The choice of *k* = 16 is empirically determined to balance computational efficiency and local context modeling. The geometric feature extraction branch produces 64-dimensional point-wise embeddings, while the RGB branch, based on MobileNetV3-small, generates 128-dimensional visual feature representations. Specifically, features are extracted from the final convolutional layer prior to the global pooling stage, ensuring the preservation of spatial information for effective cross-modal fusion.

#### 3.3.1. Geometry Branch (Deep Learning-Based Segmentation)

The geometry branch is implemented using a dynamic graph-based feature extraction strategy inspired by DGCNN. For each point, a local neighborhood graph is constructed using *k*-nearest neighbors with *k* = 16. The geometry encoder consists of four consecutive EdgeConv layers with output dimensions of 64, 64, 128, and 256, respectively. Edge features are dynamically recomputed after each layer to progressively capture evolving local geometric relationships and semantic structures. Unlike hierarchical sampling-based architectures such as PointNet++, the proposed geometry branch follows a point-wise feature propagation strategy without explicit spatial downsampling. Intermediate features extracted from multiple EdgeConv stages are concatenated to preserve both fine-grained local structures and higher-level semantic representations before multimodal fusion.

The geometry branch is responsible for extracting structural features from point cloud data using a dynamic graph-based representation [37]. Unlike grid-based methods, this approach directly operates on irregular point sets, preserving geometric fidelity. A graph *G* = (*V*, *E*) is constructed where *V* represents points, *E* represents edges formed via *k*-nearest neighbors. For each edge, a feature vector is computed as in Equation (5):

(5) $$e_{ij}=h_{\theta }(p_{i},{\;p}_{j}-p_{i}),$$

This formulation encodes absolute position $p_{i}$ and relative spatial relationship $p_{j}-p_{i}$. The use of relative coordinates ensures translation invariance, allowing the model to generalize across different spatial configurations. Feature aggregation is performed using max pooling in Equation (6):

(6) $$f_{i}=\underset{j\in N_{k}(i)}{\max}e_{ij},$$

This operation selects the most informative neighbor contribution, enhancing robustness to noise and outliers. As a result, the geometry branch effectively captures local structural patterns, which are essential for semantic segmentation.

#### 3.3.2. RGB Branch (Visual Feature Extraction)

The RGB branch extracts visual features from aligned image data using a lightweight convolutional backbone based on MobileNetV3. This branch is specifically designed to complement geometric information by incorporating appearance-based cues. The feature extraction process is defined as in Equation (7):

(7) $$g_{i}=CNN\left(I_{i}\right),$$

where $I_{i}$ denotes the image patch corresponding to point $p_{i}$. Unlike heavy backbone networks, MobileNetV3 provides an efficient trade-off between computational cost and representational power. The extracted features capture: Color variations, texture patterns, and material properties. These cues are particularly important for distinguishing classes that are geometrically similar but visually distinct, such as “cabinet” and “refrigerator”.

#### 3.3.3. Cross-Modal Graph Attention (CMGA)

To enable cross-modal feature interaction, geometric and RGB embeddings are first projected into a shared latent space using lightweight multilayer perceptrons (MLPs). Specifically, query, key, and value representations are defined as *Q_i_* = *W_q_ f_i_*, *K_j_* = *W_k_ g_j_*, and *V_j_* = *W_v_ g_j_*, where *f_i_* and *g_j_* denote geometric and RGB feature embeddings, respectively, and *W_q_*, *W_k_*, and *W_v_* are learnable projection matrices. Lightweight two-layer MLPs are used for modality-specific feature projection prior to scaled dot-product attention computation defined in Equation (8):

(8) $$\alpha_{ij}=\frac{exp(\frac{Q_{i}^{T}K_{j}}{\sqrt{d}})}{\sum_{k∊N(i)}exp(\frac{Q_{i}^{T}K_{k}}{\sqrt{d}})},$$

where *d* denotes the embedding dimension and *N*(*i*) represents the local neighborhood of point *i*. The fused representation is subsequently obtained in Equation (9):

(9) $$h_{i}=f_{i}+\sum_{j∊N(i)}\alpha_{ij}V_{j},$$

The fused representation *h_i_* combines local geometric structure with adaptively weighted RGB contextual information. This formulation enables geometry-guided RGB feature aggregation while preserving neighborhood-level spatial consistency. Finally, the combined feature is obtained as in Equation (10):

(10) $$F_{i}=f_{i\;}{+\;h}_{i},$$

This additive fusion strategy preserves the underlying geometric structure while enriching point-wise representations with context-aware RGB information obtained through cross-modal attention.

In practice, the proposed CMGA module performs geometry-guided RGB feature aggregation, where geometric embeddings act as query representations, while RGB embeddings serve as key-value representations. Although both modalities contribute to the final fused representation, the attention mechanism is not implemented as a fully symmetric bidirectional transformer-style fusion module. Therefore, the current implementation corresponds to a geometry-guided unidirectional cross-modal attention mechanism rather than a fully bidirectional fusion strategy.

Following cross-modal fusion, the resulting point-wise representations are refined through a hierarchical feature aggregation module designed to jointly capture local geometric structures and global scene-level context. First, intermediate features extracted from multiple EdgeConv stages are concatenated to preserve multi-scale neighborhood information. The aggregated features are then processed using shared multilayer perceptrons (MLPs) for feature refinement. To incorporate global contextual information, a global max-pooling operation is applied over all point features to generate a scene-level descriptor. This global representation is subsequently broadcast and concatenated back to point-level embeddings, enabling the network to enhance semantic consistency across spatially distant but semantically related regions.

#### 3.3.4. Long-Tail (LT) Learning Module

In large-scale indoor 3D semantic segmentation datasets, such as ScanNet++, the class distribution typically follows a long-tail pattern, where a small number of dominant classes (e.g., wall, floor) account for the majority of samples, while a large number of fine-grained object categories (e.g., keyboard, bottle, marker) are severely underrepresented. This imbalance leads to biased model training, where the learned decision boundaries are skewed toward frequent classes, resulting in poor generalization for rare categories [38]. Conventional deep learning models trained with standard cross-entropy loss tend to minimize overall error by prioritizing dominant classes, which further exacerbates the imbalance problem. To address this issue, we introduce a Long-Tail Learning Module that combines prototype-based representation learning with a class frequency-aware loss function. This dual strategy aims to simultaneously enhance feature separability and rebalance the learning process. The integration of prototype learning and frequency-aware weighting provides complementary benefits:

- Prototype learning: Improves feature structure and separability.
- Weighted loss: Improves training balance and gradient contribution.

Together, these components enable the model to effectively learn from highly imbalanced data distributions, leading to improved segmentation performance, particularly for rare and fine-grained object classes.

##### Prototype-Based Representation Learning

The first component of the proposed module focuses on structuring the feature space such that samples belonging to the same class are clustered around a representative center, referred to as a class prototype [39]. Formally, the prototype for class *c* is defined as in Equation (11):

(11) $$\mu_{c}=\frac{1}{\left|C\right|}\sum_{i\epsilon C}F_{i},$$

where $F_{i}$ denotes the fused feature representation of point *i* and *C* represents the set of all points belonging to class *c*. This formulation computes the centroid of class-specific features in the embedding space. The use of prototypes introduces a global structural constraint on feature learning, encouraging intra-class compactness while implicitly promoting inter-class separability. To further enforce this structure, the distance between a sample and its corresponding class prototype is defined as in Equation (12):

(12) $$d_{ic}={\left\|F_{i}-\mu_{c}\right\|}^{2},$$

Minimizing this distance ensures that features belonging to the same class are tightly clustered. This is particularly beneficial for rare classes, where limited samples may otherwise lead to fragmented or poorly defined feature distributions. By anchoring these samples to a shared prototype, the model can learn more stable and discriminative representations even under severe data scarcity.

##### Class Frequency-Aware Loss Function

While prototype learning improves feature organization, it does not directly address the imbalance in sample contributions during training. To mitigate this, we introduce a class frequency-aware weighting scheme that adjusts the loss contribution of each class based on its occurrence frequency [40]. The weight assigned to class *c* is defined in Equation (13):

(13) $${⍵}_{c}=\frac{1}{log(1+f_{c})},$$

where $f_{c}$ denotes the number of samples belonging to class *c*. This logarithmic formulation prevents extreme weight values while still providing meaningful emphasis on rare classes. The weighted segmentation loss is then expressed in Equation (14):

(14) $$L_{seg}=\sum_{i}{⍵}_{yi}\cdot l(y_{i},{\hat{y}}_{i}),$$

where $y_{i}$ is the ground-truth label, ${\hat{y}}_{i}$ is the predicted probability, l(⋅) denotes the cross-entropy loss. This formulation ensures that:

- Rare classes contribute more strongly to the loss.
- Frequent classes are prevented from dominating training.

As a result, the model achieves a more balanced optimization process, improving recall and precision for underrepresented categories.

#### 3.3.5. Segmentation Head and Objective Function

Following the multimodal feature fusion and long-tail aware representation learning, the final stage of the proposed framework is responsible for mapping the learned feature representations to semantic labels [41]. This stage consists of a lightweight segmentation head and a composite objective function designed to jointly optimize classification accuracy and feature discriminability. Unlike conventional approaches that rely solely on classification loss, the proposed framework incorporates both point-wise prediction loss and prototype-guided regularization, ensuring that the learned feature space remains well-structured while achieving high segmentation accuracy.

Segmentation Head: This is implemented as a multilayer perceptron (MLP) that operates on the fused feature representation $F_{i}$. The predicted class probabilities are obtained as in Equation (15):

(15) $${\hat{y}}_{i}=Softmax\left(MLP\left(F_{i}\right)\right),$$

here:

- The MLP acts as a nonlinear classifier that maps high-dimensional features to class logits.
- The Softmax function converts these logits into normalized probability distributions over all classes.

This design allows the model to produce per-point semantic predictions, ensuring fine-grained segmentation across the entire scene.

The segmentation head consists of a three-layer MLP with output dimensions of 256, 128, and *C*, respectively, where *C* denotes the number of semantic classes. Each intermediate layer is followed by Batch Normalization, ReLU activation, and dropout with a rate of 0.3. After multimodal fusion, the hidden feature dimension is set to 256. Prototype representations are updated during training using exponential moving average updates with a momentum coefficient of 0.9. The overall loss function is formulated in Equation (16):

(16) $$L_{total}={\lambda_{1}L}_{seg}+{\lambda_{2}L}_{proto},$$

where $\lambda_{1}=1.0$ and $\lambda_{2}=0.5$ balance the segmentation and prototype-guided losses, respectively. $L_{seg}$ is the class frequency-aware segmentation loss, $L_{proto}$ is the prototype consistency loss, λ is a balancing hyperparameter.

Segmentation Loss: As defined earlier in Equation (14). This term ensures accurate classification while accounting for class imbalance.

Prototype Consistency Loss: The prototype loss enforces alignment between feature representations and their corresponding class centers is calculated in Equation (17):

(17) $$L_{proto}=\sum_{i}{\left\|F_{i}-\mu_{yi}\right\|}^{2},$$

This term encourages compact intra-class distributions and clear separation between classes.

Optimization Perspective: From an optimization standpoint:

- $L_{seg}$ guides the model toward correct predictions.
- $L_{proto}$ regularizes the feature space.

The balancing parameter λ controls the trade-off between these objectives. A higher value of λ emphasizes feature clustering, while a lower value prioritizes classification accuracy.

As a result the proposed objective function enables the model to achieve high segmentation accuracy, maintain a structured and interpretable feature space, and improve generalization under long-tail distributions. By jointly optimizing classification and representation learning, the model effectively addresses both data imbalance and feature ambiguity, which are the primary challenges in fine-grained 3D semantic segmentation.

## 4. Experimental Results

**Figure 3:**
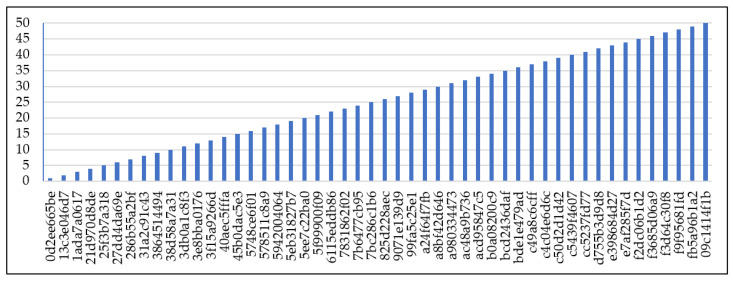
Visualization of the ScanNet++ 50-scene subset id used in the experimental evaluation.

The ScanNet++ dataset is distributed under an access-controlled protocol. The subset used in this study corresponds to the portion officially provided through the dataset access request process, consisting of 50 indoor scenes with associated mesh and semantic annotations. To avoid selection bias, all accessible scenes were included in the experiments without additional filtering or manual scene exclusion. Scene-level splitting was applied to avoid spatial overlap between training and testing samples. The dataset was partitioned at the scene level into training (35 scenes), validation (5 scenes), and testing (10 scenes) subsets using a fixed split protocol. The identifiers of the 50 ScanNet++ subset scenes used in the experimental evaluation are presented in Figure 3. The visualization provides the exact scene allocation employed throughout the training, validation, and testing stages to improve experimental transparency and reproducibility.

To rigorously evaluate the effectiveness of the proposed LT-CM-GACNet++ framework, extensive experiments are conducted on the ScanNet++ dataset, which provides high-resolution indoor scenes with rich geometric and RGB information. The dataset presents a particularly challenging benchmark due to its fine-grained semantic classes and long-tail distribution, making it suitable for assessing both segmentation accuracy and robustness under class imbalance. The dataset is split at the scene level into training, validation, and test subsets consisting of 35, 5, and 10 scenes, respectively (70%/10%/20%). The class distribution exhibits a pronounced long-tail characteristic, where the top-10 most frequent classes account for approximately 75–80% of the total points, while more than 50 classes each contribute less than 1% of the data. This scene-level partitioning prevents spatial overlap between splits and effectively eliminates information leakage, ensuring a fair and unbiased evaluation. All point clouds are preprocessed using the pipeline described in Section 3.2, including density-aware sampling, normal estimation, and block partitioning with a fixed size of *N* = 1024 points per block. The input features consist of 3D coordinates (*x*, *y*, *z*), surface normals (*n*_*x*_, *n*_*y*_, *n*_*z*_), and RGB color values (*r*, *g*, *b*). The model is implemented in PyTorch 2.1.0 and trained using the AdamW optimizer with an initial learning rate of 1 × 10^−3^, cosine decay scheduling, and a weight decay of 0.01. The batch size is set to 16, and training is conducted for 200 epochs. The loss weights are empirically set as *λ*_1_ = 1.0 and *λ*_2_ = 0.5. Early stopping is applied based on validation performance to prevent overfitting.

### 4.1. Quantitative Evaluation

To provide a comprehensive performance analysis, multiple evaluation metrics are employed: Mean Intersection over Union (mIoU): Measures overall segmentation quality. Accuracy (Acc): Evaluates correct predictions. Precision, Recall, and F1-score: Assess classification balance. Rare-class mIoU (mIoU_rare): Evaluates performance on underrepresented classes. The inclusion of mIoU_rare is particularly important, as it directly reflects the effectiveness of the proposed long-tail learning strategy.

To quantitatively evaluate the performance of the proposed LT-CM-GACNet++ model, extensive experiments were conducted on the ScanNet++ dataset. The evaluation protocol follows widely adopted semantic segmentation metrics, including mean Intersection over Union (mIoU), classification accuracy (Acc), precision, recall, and F1-score. In addition, to better assess the effectiveness of the proposed framework under long-tail distributions, rare-class mIoU is also reported. The proposed approach is compared with several recent and widely adopted semantic segmentation methods in terms of both overall segmentation performance and its ability to distinguish underrepresented classes. The detailed quantitative comparison results are presented in Table 2.

The results in Table 2 clearly demonstrate that the proposed LT-CM-GACNet++ achieves superior performance across all evaluation metrics. In particular, the model outperforms the strongest baseline (PTv3) by +4.1% mIoU and +7.6% mIoU_rare. This improvement is not only a consistent performance improvement but also methodologically meaningful. While transformer-based models such as PTv3 are effective at capturing global contextual relationships, they lack explicit mechanisms for handling class imbalance. As a result, their performance gains are primarily concentrated on dominant classes. In contrast, the proposed method explicitly integrates long-tail learning through both feature-level (prototype learning) and loss-level (class weighting) strategies. This dual approach leads to substantial improvements in rare-class recognition without sacrificing overall performance. Furthermore, the improvement in F1-score indicates that the model achieves a better balance between precision and recall, suggesting more reliable predictions across diverse semantic categories.

**Figure 4:**
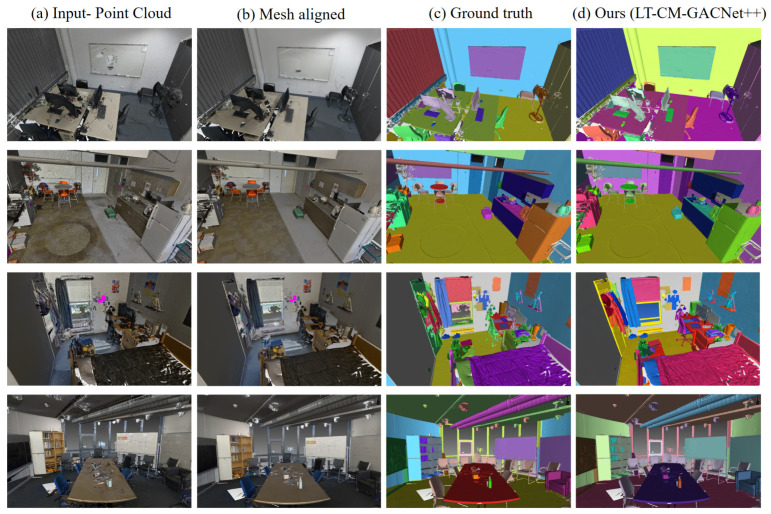
Qualitative semantic segmentation results on ScanNet++ scenes: (**a**) shows the input point clouds, (**b**) presents mesh-aligned scene representations, (**c**) corresponds to ground-truth annotations, and (**d**) illustrates segmentation predictions generated by the proposed LT-CM-GACNet++ framework. “Mesh-aligned” refers to the spatially registered textured mesh reconstructed from RGB-D observations.

Qualitative segmentation result samples on the ScanNet++ dataset are shown in Figure 4. As illustrated in Figure 4, the proposed LT-CM-GACNet++ framework produces segmentation boundaries that are visually more coherent and spatially smoother while remaining highly consistent with the ground-truth annotations.

### 4.2. Ablation Study

To systematically evaluate the contribution of each component in the proposed framework, a comprehensive ablation study is conducted. Performance results for component-wise performance improvement are given in Table 3. The implementation without CMGA, also defined as + RGB Branch, refers to RGB features extracted from MobileNetV3-small that are directly concatenated with geometric features without applying CMGA. The ablation results clearly indicate that the largest performance gain is achieved by the long-tail learning module, confirming its effectiveness in handling class imbalance.

As a result of multimodal integration, the introduction of the RGB branch leads to a +3.3% improvement in mIoU, confirming that visual features provide complementary information to geometric representations. This is particularly important for distinguishing objects with similar shapes but different appearances. The CMGA module contributes an additional +1.8% improvement, highlighting the importance of adaptive feature fusion. Unlike simple concatenation, the attention mechanism selectively emphasizes informative features, leading to more robust representations. The inclusion of class frequency-aware loss results in a significant improvement in rare-class performance (+4.8%) and in mIoU (+1.9%), demonstrating its effectiveness in mitigating class imbalance during training. Finally, prototype-based learning further improves mIoU_rare by +4.2%, confirming that structuring the feature space is essential for fine-grained classification. This component plays a crucial role in reducing intra-class variance and improving decision boundaries for rare classes.

**Figure 5:**
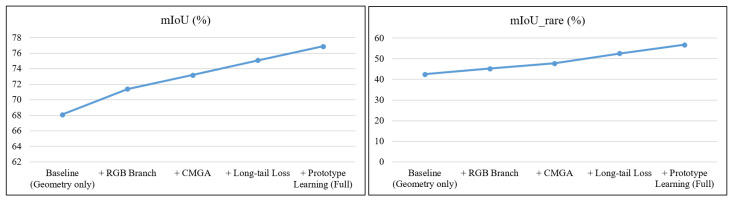
Component-wise performance analysis of the proposed framework. The graph illustrates the incremental improvements in mIoU and mIoU_rare obtained by progressively integrating RGB fusion, CMGA, and long-tail aware learning modules.

Figure 5 illustrates the component-wise performance improvement of LT-CM-GACNet++, showing that each module progressively enhances segmentation accuracy. The CMGA module improves cross-modal contextual learning, multimodal fusion strengthens semantic representation, and the long-tail learning strategy further boosts the discrimination of underrepresented classes, resulting in the highest mIoU performance.

To provide a comprehensive evaluation of the computational efficiency of the proposed architecture, an end-to-end performance analysis was conducted by measuring the execution time of each processing stage, including pre-processing, geometry and RGB feature extraction branches, CMGA-based fusion, and the segmentation head, with the aggregated results summarized in Table 4. The results show that the proposed framework maintains a reasonable computational cost despite its architectural complexity. The use of MobileNetV3 ensures that the RGB branch remains efficient, while the graph-based operations are optimized through neighborhood sampling.

To evaluate experimental stability, each experiment was repeated three times using different random seeds. The proposed LT-CM-GACNet++ framework achieved an average mIoU of 76.9% with a standard deviation of ±0.4, indicating stable segmentation performance across repeated runs.

Runtime evaluation was conducted on a workstation equipped with an NVIDIA RTX 4060 GPU and an Intel i7 processor. The execution time reported in Table 4 corresponds to the pure forward-pass inference time of the segmentation network for a single scene block, excluding preprocessing operations. In contrast, the end-to-end processing time reported in Table 5 additionally includes preprocessing stages such as density-aware sampling, normal estimation, and block generation, resulting in an average total processing time of approximately 1.7 s per frame-equivalent block.

**Figure 6:**
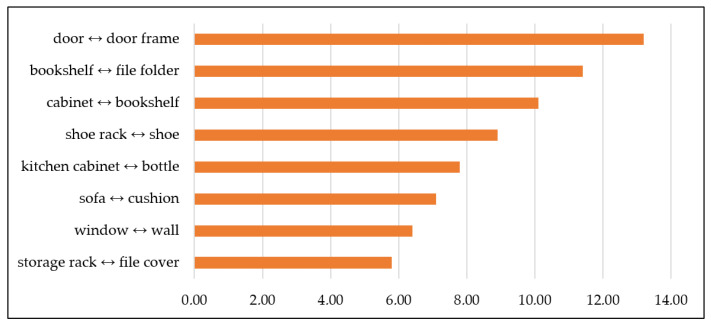
Rare-class confusion matrix analysis for the proposed LT-CM-GACNet++ model.

To assess the effectiveness of the proposed method in comparison with existing approaches, a comprehensive analysis was conducted by jointly considering segmentation accuracy and computational efficiency, where performance is evaluated in terms of both overall mIoU and rare class mIoU alongside the corresponding inference time, as presented in Table 5. Table 4 reports the forward inference time of the segmentation network only, whereas Table 5 includes the complete end-to-end pipeline, including preprocessing and multimodal feature extraction stages. The results indicate that the proposed approach achieves higher mIoU and improved performance on rare classes while maintaining a competitive processing time. Table 5 highlights the trade-off between performance and computational efficiency. While transformer-based models achieve strong accuracy, they incur higher computational costs. The proposed method strikes a balanced trade-off by combining efficient CNN-based feature extraction with graph-based learning. The misclassification distribution among visually and geometrically similar rare classes in the ScanNet++ evaluation subset is illustrated in Figure 6.

**Figure 7:**
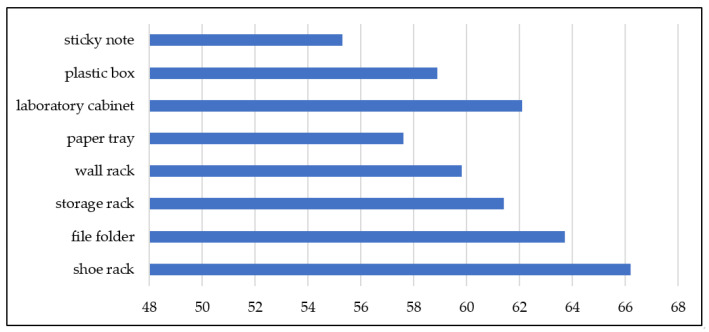
Class-wise IoU% comparison for representative underrepresented categories in the ScanNet++ evaluation subset.

Figure 6 shows that most classification ambiguities occur between semantically related indoor categories with highly similar geometric structures and contextual appearances. In particular, confusion is more frequently observed between pairs such as door–door frame, bookshelf–file folder, cabinet–bookshelf, and shoe rack–shoe. These categories often exhibit overlapping spatial layouts, similar textures, or close geometric proximity within indoor environments, making fine-grained segmentation substantially more challenging. Nevertheless, the relatively reduced confusion rates indicate that the proposed CMGA-based multimodal fusion strategy effectively improves feature discrimination by jointly utilizing geometric neighborhood information and RGB contextual cues. Furthermore, the prototype-guided long-tail learning mechanism contributes to reducing feature overlap among underrepresented categories. The class-wise IoU performance of representative rare categories obtained by the proposed LT-CM-GACNet++ framework is presented in Figure 7.

As illustrated in Figure 7, the proposed framework achieves relatively stable segmentation performance across several low-frequency indoor object categories despite the severe long-tail distribution characteristics of the ScanNet++ subset. Categories such as shoe rack, file folder, storage rack, and wall rack demonstrate comparatively strong IoU performance, indicating that the proposed prototype-based representation learning strategy effectively enhances minority-class feature separability. Although certain categories with highly limited training samples still exhibit moderate performance degradation, the overall results confirm that the integration of cross-modal attention and class frequency-aware optimization substantially improves rare-class recognition capability. These findings further validate the robustness of the proposed LT-CM-GACNet++ framework for fine-grained indoor semantic segmentation under long-tail distributions.

## 5. Discussion

The proposed LT-CM-GACNet++ framework demonstrates several important advantages for fine-grained 3D semantic segmentation under long-tail class distributions. First, the integration of dynamic graph-based geometric feature extraction with RGB-based visual representations enables more discriminative multimodal feature learning compared to geometry-only approaches. This complementary fusion strategy contributes to consistent improvements in both overall segmentation accuracy and rare-class performance on the ScanNet++ benchmark subset. Second, the proposed Cross-Modal Graph Attention (CMGA) module improves the interaction between geometric and visual modalities through adaptive feature weighting. By facilitating information exchange across modalities, the framework captures both local geometric structures and semantic appearance cues more effectively. Experimental results indicate that this design improves segmentation consistency, particularly for visually similar indoor objects and structurally complex regions. Third, the long-tail learning strategy based on prototype-guided representation learning and class frequency-aware loss weighting improves feature separability for underrepresented categories. The observed gains in rare-class mIoU demonstrate that explicitly addressing class imbalance is beneficial for fine-grained indoor scene segmentation.

The preprocessing pipeline, including density-aware sampling, normal estimation, and block partitioning, is mainly designed to provide stable and computationally consistent inputs for training and inference. Although these preprocessing steps contribute to efficient data preparation, their individual effects were not independently evaluated through dedicated ablation experiments. Therefore, their impact should be interpreted as supportive preprocessing operations rather than standalone methodological contributions. Future work will investigate the influence of alternative preprocessing and sampling strategies through additional controlled experiments.

In addition to point-cloud-only baselines, recent multimodal segmentation frameworks such as MIT [43] were also considered during analysis. Unlike conventional point-based architectures, MIT performs explicit 2D–3D interlaced feature fusion using transformer-based attention mechanisms. Compared to such multimodal approaches, the proposed LT-CM-GACNet++ framework can achieve competitive segmentation performance while maintaining lower architectural complexity and improved rare-class discrimination through the proposed long-tail learning strategy. Moreover, the preprocessing pipeline, including density-aware sampling, normal vector estimation, and block-based partitioning, ensures consistent and efficient data representation. These steps contribute to stabilizing the training process and improving the model’s generalization capability across scenes with varying point densities and structural complexity. Compared to methods that rely on fixed or uniform sampling strategies, the proposed preprocessing approach better preserves geometric fidelity while maintaining computational tractability.

Although the proposed framework demonstrates strong performance on the accessible ScanNet++ subset, evaluation on the complete benchmark dataset may further validate the generalization capability and scalability of the proposed architecture across a wider variety of indoor scene configurations.

It should be noted that the experimental evaluation was conducted using the accessible 50-scene subset provided through the official ScanNet++ access procedure. Although this subset contains diverse indoor environments and severe long-tail characteristics, it remains smaller than the complete benchmark commonly used in large-scale evaluations. To ensure fairness, all baseline methods were retrained under identical preprocessing, optimization, and evaluation settings using the same subset partition. Nevertheless, the restricted dataset size may limit the generalizability of the reported performance comparisons. Future work will therefore focus on extending the evaluation to larger benchmark splits and additional indoor 3D scene understanding datasets.

Although the proposed framework demonstrates competitive performance, direct comparison with recent multimodal architectures remains limited due to differences in dataset accessibility, evaluation protocols, and implementation settings. Future work will include broader multimodal benchmark comparisons under standardized experimental conditions.

Despite these strengths, several limitations of the proposed framework should be acknowledged. First, although the CMGA module improves cross-modal feature interaction, it introduces additional computational complexity, particularly for large-scale scenes with high point densities. While the lightweight MobileNetV3 backbone partially reduces this overhead, further optimization is necessary for deployment in real-time or resource-constrained applications. Second, although the long-tail learning strategy improves rare-class recognition, classes with extremely limited samples remain challenging. In such cases, prototype representations may not fully capture intra-class variability, potentially limiting generalization performance. Future studies may explore meta-learning or adaptive reweighting strategies to further enhance minority-class learning. In addition, the current multimodal fusion framework assumes reliable alignment between geometric and RGB modalities. In practical scenarios, sensor noise, inaccurate calibration, or missing visual observations may reduce segmentation robustness. Developing more robust cross-modal alignment and modality-adaptive fusion strategies, therefore, remains an important research direction. Furthermore, although the proposed framework demonstrates strong performance on the ScanNet++ benchmark subset, its generalization capability across datasets with different sensing characteristics, such as outdoor LiDAR environments, has not yet been investigated. Extending the framework toward cross-dataset adaptation and broader scene generalization constitutes an important avenue for future work.

The integration of the RGB feature extraction branch and the CMGA fusion module introduces additional computational overhead during training compared to geometry-only segmentation frameworks. In particular, multimodal feature extraction and graph-attention operations increase GPU memory consumption and training time due to the simultaneous processing of geometric and visual representations. However, the use of the lightweight MobileNetV3-small backbone significantly reduces this overhead compared to heavier CNN encoders. Experimental observations indicate that the additional computational cost remains manageable on consumer-grade GPUs while providing substantial gains in segmentation accuracy and rare-class recognition performance.

Overall, the proposed LT-CM-GACNet++ model introduces a unified and effective solution for multimodal 3D semantic segmentation under long-tail distributions. By combining cross-modal attention, efficient feature extraction, and imbalance-aware learning, the framework demonstrates the effectiveness of multimodal feature integration for fine-grained indoor semantic segmentation under long-tail distributions while also highlighting several directions for future improvement.

## 6. Conclusions

This study presents a novel Long-Tail Aware Cross-Modal Graph Attention Network (LT-CM-GACNet++) for fine-grained 3D semantic segmentation of high-resolution indoor point cloud data. The proposed framework is specifically designed to address the key challenges of multimodal feature integration, class imbalance, and complex geometric structures commonly encountered in datasets such as ScanNet++. By effectively combining dynamic graph-based geometric feature extraction with a lightweight RGB-based backbone, the model achieves a balanced trade-off between computational efficiency and segmentation accuracy. A central contribution of this work is the proposed Cross-Modal Graph Attention (CMGA) module, which enables adaptive and geometry-guided cross-modal feature interaction between geometric and visual modalities. This mechanism significantly enhances the model’s ability to capture both local geometric details and global contextual relationships. In addition, the integration of a long-tail aware learning strategy, based on prototype-guided representation learning and class frequency-aware loss, improves the recognition performance of underrepresented classes while maintaining strong overall segmentation performance. Extensive experimental evaluations conducted on the ScanNet++ dataset demonstrate that the proposed method consistently outperforms existing approaches in terms of both overall mIoU and rare-class mIoU metrics. The results confirm that the synergistic integration of multimodal learning, attention mechanisms, and imbalance-aware optimization leads to more robust and discriminative feature representations. Furthermore, the ablation studies highlight the individual contributions of each component, validating the effectiveness of the proposed design choices. Despite its strong performance, the study also identifies several areas for future improvement. These include reducing the computational overhead of cross-modal attention mechanisms, enhancing robustness to extreme class imbalance and noisy data, and improving generalization across different datasets and sensing modalities. Addressing these challenges will be essential for deploying such models in real-world applications, particularly in resource-constrained or dynamic environments. In conclusion, this work demonstrates that cross-modal learning combined with long-tail aware optimization provides a powerful and scalable solution for high-resolution 3D semantic segmentation. The proposed LT-CM-GACNet++ framework provides an effective and flexible foundation for future research in multimodal 3D scene understanding and intelligent spatial analysis systems.

## Figures and Tables

**Table 1:** Statistical summary of the selected ScanNet++ subset used in the experiments.

Property	Value
Number of Scenes	50
Number of Classes	~100
Data Type	Point Cloud + RGB + Mesh
Input Features	XYZ + RGB + Normal
Average Points per Scene	~1M+
Characteristics	High-resolution, multimodal
Key Challenge	Long-tail distribution

**Table 2:** Segmentation performance comparison on the ScanNet++ evaluation subset.

Method	mIoU (%)	mIoU_rare (%)	Acc (%)	Prec (%)	Rec (%)	F1 (%)
PointNet++ [16]	62.4	38.7	81.2	76.1	70.4	73.1
DGCNN [42]	65.8	41.3	83.6	78.9	72.6	75.6
Point Transformer [18]	69.5	45.6	85.1	81.2	75.8	78.4
PTv3 [20]	72.8	49.2	87.3	83.6	78.4	80.9
Ours	76.9	56.8	89.1	85.7	81.3	83.4

**Table 3:** Ablation analysis of the proposed LT-CM-GACNet++ components on the ScanNet++ evaluation subset.

Configuration	mIoU (%)	mIoU_Rare (%)
Baseline (Geometry only)	68.1	42.5
+ RGB Branch	71.4	45.2
+ CMGA	73.2	47.8
+ Long-tail Loss	75.1	52.6
+ Prototype Learning (Full)	76.9	56.8

**Table 4:** Pure network inference time comparison.

Stage	Time (s)
Pre-processing	0.05
Geometry Branch	0.42
RGB Branch	0.31
CMGA Fusion	0.18
Segmentation Head	0.09
Total	1.05 s/frame

**Table 5:** End-to-end execution time including preprocessing stages.

Method	mIoU (%)	mIoU_Rare (%)	Time (s)
DGCNN	65.8	41.3	1.2
PTv3	72.8	49.2	2.1
Ours	76.9	56.8	1.7

## Data Availability

All relevant data are fully available within the manuscript without restriction. The ScanNet++ data that support this study’s conclusions are publicly available online: https://scannetpp.mlsg.cit.tum.de/scannetpp (accessed on 17 April 2026).
